# Sensors Based on Optical and Photonic Devices

**DOI:** 10.3390/s26020726

**Published:** 2026-01-21

**Authors:** Francesco De Leonardis

**Affiliations:** Department of Electrical and Information Engineering, Politecnico di Bari, Via Orabona, 4, 70126 Bari, Italy; francesco.deleonardis@poliba.it

## 1. Introduction

Programmable photonics is an emerging technology that merges photonics and electronics, enabling innovative light-based information processing with high speed and low power consumption. In this context, the increasing maturity of integrated photonic technologies now allows for the development of larger and more complex photonic circuits on a single chip [1,2,3,4]. A new generation of software-programmable photonic circuits, featuring on-chip waveguide meshes, tunable beam couplers, and optical phase shifters, has been developed to address growing demands in functionality and complexity. Fully integrated programmable photonic devices have potential applications across a wide range of fields, including data centers, precision sensing, artificial intelligence (AI), biomedicine, on-chip single-photon manipulation, multiphoton microscopy, and quantum computing.

From this perspective, photonic sensors play a pivotal role in driving innovation and growth. Their immunity to electromagnetic interference (EMI) makes them ideal for environments with high EMI levels, such as power plants and industrial facilities [5,6,7]. One of their main advantages lies in their ability to deliver highly sensitive [8,9,10], fast, and accurate measurements across diverse applications. In healthcare, photonic sensors are employed in medical imaging technologies, such as optical coherence tomography, for non-invasive diagnostics and tissue imaging. Additionally, they have enhanced environmental monitoring by enabling real-time detection of pollutants, gases, and other substances in air and water. Their applications also extend to defense and security, where they are used in surveillance, reconnaissance, and laser-based defense systems.

Photonic sensors work by converting variations in light into electronic signals, offering readable and quantitative data [11]. Thanks to advancements in electronics, these sensors can now be integrated into portable platforms such as lab-on-a-chip systems, lateral-flow assays, smart textiles, food packaging, various wearable devices [12,13,14], and even biological systems. However, challenges remain in developing sensors with high analytical performance, due to current technological constraints and limitations inherent to sensing principles [15]. Future research will focus on miniaturization, integration with complementary technologies such as AI [16] and nanotechnology, and performance enhancement through the use of novel materials.

## 2. Overview of Published Papers

All submissions were evaluated based on their technical merit and relevance, and twelve high-quality papers were ultimately accepted for inclusion in this Special Issue.

Below, we present the list of accepted contributions, each followed by a brief summary.

In contribution 1, the authors conducted both numerical and experimental investigations of stimulated Brillouin scattering (SBS) in highly birefringent spun optical fibers. They observed a periodic oscillation of the Brillouin gain induced by fiber bending. Experimental results confirmed that the Brillouin gain in bent spun fibers displays periodic undulations with a period corresponding to the elliptical beat length of the spun fiber. This finding enables the recovery of fiber birefringence using conventional Brillouin measurements. The study also analyzed the formation mechanism of these undulations through numerical simulations of the state-of-polarization (SOP) evolution. The authors concluded that spun fibers are suitable for simultaneous strain and temperature sensing.

In contribution 2, a novel method was proposed for eliminating 1/f noise of optical origin using a micro-cavity Fabry–Pérot (FP) interferometer. Mechanical modulation of the FP cavity length was applied to a previously developed opto-mechanical sensor, effectively mimicking laser frequency up-conversion. This shifts the signal to a frequency region where white noise dominates, and 1/f noise is absent. The approach is compact, cost-effective, and does not require external electro-optic components such as EOMs or AOMs. The system achieved cavity frequency modulations up to 1 THz with low mechanical modulation near 500 kHz, and performance not achievable with other techniques. This modulation strategy offers a practical method for enhancing displacement sensitivity in homodyne readout systems, with potential applications in displacement, refraction, and spectroscopy measurements.

In contribution 3, the authors developed a digital holography-based technique to detect the vacuum level in vacuum glass, an essential parameter that reflects the quality and performance of such glass. The system comprises an optical pressure sensor, a Mach–Zehnder interferometer, and custom software. The study demonstrated that deformation in a monocrystalline silicon film responds to vacuum degradation. Experiments under different vacuum conditions validated the system’s ability to provide fast and accurate measurements. Moreover, adjusting the optical pressure sensor’s film parameters enables improvements in measurement range and accuracy, highlighting the technology’s market potential.

In contribution 4, a novel Fiber Loop Mirror (FLM) configuration incorporating two circulators was presented and characterized for sensing applications. The design supports strain and torsion discrimination and enables the use of varying fiber lengths and refractive indices. This allows control of the free spectral range (FSR) and generation of birefringence values exceeding 10^−2^, which is critical for developing highly sensitive sensors leveraging the Vernier effect. The ability to achieve low FSR with short fiber lengths enhances strain sensitivity via a push–pull method.

In contribution 5, the authors explored gradient strain field measurements using both point fiber-optic sensors based on Bragg gratings and distributed sensors using Rayleigh scattering. Various sample geometries were designed to induce different strain gradient distributions under uniaxial tension, including those with changing derivative signs. Samples were created from polymer composites (via direct pressing) and 3D-printed polymers with embedded sensors. Simulations were conducted to assess the error in strain estimation using data from point and distributed sensors.

In contribution 6, the authors assessed the suitability of ultra-compact microspectrometers (C12880MA and C11708MA) for use in portable spectroscopic devices with spatial resolution. Their potential was evaluated in the context of monitoring optical properties in condensed scattering media, with a focus on space-efficient integration.

In contribution 7, two Sky Quality Meter (SQM-LE) units were integrated into an autonomous sensor suite developed at the University of Padova for monitoring light pollution using drones or sounding balloons. Laboratory testing with controlled light sources revealed deviations in angular response compared to manufacturer specifications. The sensors exhibited a double-peak angular response and broader-than-expected FWHM. Orientation sensitivity was also analyzed, linking angular deviations to imperfect optical alignment. Three-dimensional sensitivity maps were generated and compared to expected models, showing that light sources near the edge of the field of view can significantly impact data accuracy, especially in stratospheric balloon applications where orientation is uncontrolled.

In contribution 8, the authors investigated the effects of seasonal temperature variations on borehole behavior and thermal gradients over time. It also highlighted how optimally sized and drilled boreholes for heat pumps can significantly reduce energy consumption compared to conventional heating systems. These insights are particularly relevant for designing borehole depths and counts in residential applications.

In contribution 9, the authors presented a comprehensive review of optical fiber sensing technologies, assessing current methods, their advantages, disadvantages, and limitations compared to competing technologies. With the 2021 IPCC report in mind, the review emphasized detecting major greenhouse gases and the challenges associated with their measurement, underscoring the relevance of optical fiber sensing in environmental monitoring.

The review of contribution 10 highlighted recent developments in biomedical photonic sensors over the past five years, focusing on fiber-optic technologies across multiple medical fields. Key trends included the use of multiparameter sensing, integrated control systems, and fiber-based probes in devices like catheters, needles, and endoscopes. Notable sensor performances for both physical and biochemical measurements were discussed.

In contribution 11, the authors explored optical techniques for measuring ocular aberrations, focusing on the pyramidal wavefront sensor (PWS) aberrometry. This method showed superior resolution in evaluating multifocal intraocular lenses (MF-IOLs), commonly used in cataract and refractive surgeries. Aberrometry enabled comparison of retinal image quality across different lens technologies. Results indicated that monofocal and diffractive lenses provided better far-distance image quality than refractive or extended depth-of-focus (EDoF) lenses. Further research is encouraged to enhance clinical decision-making regarding lens selection.

In contribution 12, the authors proposed a doctrinal framework for “Sensing using Light” (SuL), offering a unified perspective encompassing all light-based sensing approaches. Using a bottom-up methodology, the authors defined key sensing system components: the optical transducer, the optical channel, and the optoelectronic unit. Together, these elements generate accurate electrical representations of the measurand. When combined with intelligence capable of actuation, the sensor evolves into a Smart Photonic Sensor. The paper emphasizes the role of photonics, defined as the science and technology of light, in generating, propagating, controlling, and detecting optical signals for diverse applications.

## 3. Conclusions

In this Special Issue, we present twelve selected papers that explore various topics related to sensors based on optical and photonic devices. The aim is to highlight the current state of the art and future developments in this broad, evolving, and significant area of research. This collection offers an excellent opportunity to bring together the global scientific community working in photonics and related fields, showcasing original, innovative, and high-impact research. Ultimately, our goal is to provide a valuable reference for understanding current trends, identifying challenges, and exploring potential solutions in photonic technologies. I would like to extend my sincere thanks to all the authors who contributed their work to this Special Issue, as well as to the reviewers, whose time and expertise were essential in ensuring the quality and success of this publication.
